# A Cost-Effective Method for the Spectral Calibration of Photoplethysmography Pulses: The Optimal Wavelengths for Heart Rate Monitoring

**DOI:** 10.3390/s25072311

**Published:** 2025-04-05

**Authors:** Vinh Nguyen Du Le, Sophia Fronckowiak, Elizabeth Badolato

**Affiliations:** Department of Physics and Astronomy, University of Alabama in Huntsville, Huntsville, AL 35899, USA

**Keywords:** photoplethysmography (PPG), hemoglobin (Hb), optical phantoms, source–detector distance (SDD), heart rate

## Abstract

A photoplethysmography (PPG) pulse in reflection mode represents the change in diffuse reflectance at the skin surface during a cardiac cycle and is commonly used in wearable devices to monitor heart rate. Commercial PPG sensors often rely on the reflectance signal from light sources at two different wavelength regions, green, such as *λ* = 523 nm, and near infrared (NIR), such as *λ* = 945 nm. Early in vivo studies of wearable sensors showed that green light is more beneficial than NIR light in optimizing PPG sensitivity. This contradicts the common trends in the standard near infrared spectroscopy techniques, which rely on the long optical pathlengths at NIR wavelengths to achieve optimal depth sensitivity. To quantitatively analyze the spectral characteristics of PPG across the wavelength region of 500–900 nm in a controlled environment, this study performs the spectral measurement of PPG signals using a simple and cost-effective optical phantom model with two distinct layers and a customized diffuse reflectance spectroscopy system. In addition, Monte Carlo simulations are used to elaborate the underlying phenomena at the green and NIR wavelengths when considering different epithelial thicknesses and source–detector distances (SDD).

## 1. Introduction

Photoplethysmography (PPG) is a non-invasive and cost-effective optical technique that can detect blood volume changes between the systolic and diastolic phases of the cardiac cycle and is commonly used in wearable devices to monitor heart rate and to track physical activity [1,2]. Generally, blood volume increases in the systolic phase, resulting in an increase in the absorbance of light (decrease in reflectance) in tissue compared to that in the diastolic phase. Most wearable devices measure PPG in reflection mode by utilizing light sources at two different wavelengths (green at 523 nm and near infrared or NIR between 900 and 1000 nm) and multiple detectors located between 2.4 mm and 5.5 mm from the sources [3]. Using non-linear time series analysis for the chaotic analysis of reflectance PPG from finger PPG sensors, early studies showed that both green PPG and NIR PPG are chaotic, while the green PPG dynamics are more complex than those of NIR PPG, and both can be used in different applications, including heart rate and mental and physiological health monitoring [4]. Other in vivo studies also showed that green PPG correlates better with electrocardiogram (ECG) than NIR PPG [5,6] and that green PPG produces higher-diastolic-pressure components than NIR PPG during heat stress [7]. Though the effects of detector geometries, source-detector distances (SDD), skin anatomy, and skin tones were not studied in these earlier experimental studies, they have been investigated using computational models [3,8,9,10]. Focusing on long source–detector distances (SDD > 2.0 mm), Ajmal et al. showed that NIR light underperforms in obese individuals, yielding a lower PPG signal than that of green light [3]. They argued that the reduced absorbance of blood within the vessels in the NIR range lead to this poor performance. Simulating a layer of blood in skin, Laipitan et al. showed that green PPG is significantly better than NIR PPG in most cases [8]. In our previous studies, the simulations also showed a similar trend [9], which contradicts the common practice of standard NIR spectroscopy in studying brain activities, where better brain activation is known to be associated with long partial pathlengths in the brain at NIR wavelengths [10,11,12].

In this paper, we perform PPG pulses measurement in the wavelength region of 450–900 nm on two-layered hemoglobin-based phantoms using a customized diffuse reflectance spectroscopy system. Subsequently, the dependence of PPG signal on wavelengths and epidermal thickness is observed, while the advantages of PPG at short wavelengths over PPG at NIR wavelengths is demonstrated. To investigate the role of photon partial pathlength on PPG sensitivity, we extend our previous modeling effort [9] to compute both the PPG and photon pathlengths in reflectance mode, while considering three different epidermal thicknesses and four different SDD at both the NIR and green wavelengths. This study will provide a complete guideline to improve heart rate monitoring in optical wearables.

## 2. Methods

### 2.1. Optical Phantoms for PPG Measurement

**Figure 1:**
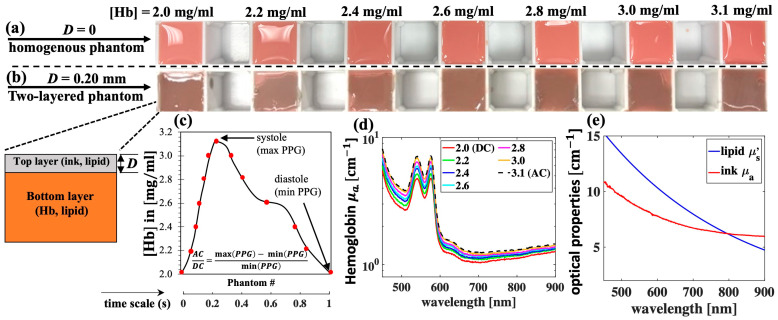
Gel-based optical phantoms with various hemoglobin (Hb) concentrations for PPG measurement. (**a**) Homogenous phantoms (*D* = 0); (**b**) two-layered phantoms with top layer thickness *D* = 0.20 mm; (**c**) Hb concentrations of phantoms are selected to simulate one PPG pulse so that each phantom represent one data point; (**d**) measured absorption coefficient, *μ*_a_, spectra of selected Hb concentrations; and (**e**) *μ*_a_ and *μ*_s_′ spectra of ink 1% *v*/*v* and lipid 1% *w*/*v*, respectively.

Though recent studies have developed novel pulsatile optical phantoms to calibrate PPG waveforms [13,14,15,16], the procedures are complicated, associated with a high cost, and often lack of the signature absorption of hemoglobin for the accurate evaluation of PPG signals over a broadband of wavelengths. Here, simple gel-based optical phantoms with two distinct layers are produced for the spectral calibration of PPG. While gel-based phantoms have widely been used to create optical phantoms, their application was limited to studying the accuracy of the inverse models in measuring layered optical properties [17,18,19,20]. Here, the PPG waveform and the AC/DC signal are the main targets. The optical phantoms are constructed with deionized water, agarose (Type VII, Sigma-Aldrich, St. Louis, MO, USA), Intralipid (I141, Sigma Aldrich, St. Louis, MO, USA), black India ink (Chartpak Inc., Leeds, MA, USA), and FDA-approved human whole blood (Innovative Research, Novi, MI, USA). The selected agar type has high gel strength and 100% transmittance [21]. The main absorber of the top layer is ink, whereas the main absorber of the bottom layer is hemoglobin (Hb). India ink is used for its similar absorption spectral shape to that of melanin found in the epidermis, whereas Intralipid is commonly used to simulate tissue scattering [21,22,23,24,25,26,27,28]. Like previous studies, the absorption coefficients (*μ*_a_) of ink and hemoglobin are calculated by applying Beer-Lambert’s law to the measured transmission using a spectrophotometer (Unico, Cole Parmer, Vernon Hills, IL, USA) [21,22], and a PPG pulse is calculated by taking the negative logarithm of diffuse reflectance [9]. To simulate the full PPG waveform (determined by the change in blood volume over a cardiac cycle), a set of seven phantoms whose bottom layers carried different hemoglobin concentrations is used (Figure 1a–c).

The production of phantoms begins with the preparation of stock gel solution with a concentration of 2.5% *w*/*v* by dissolving Agar in distilled water at 65 °C for 30 min. Concentrated Agar solution is then added to the freshly made mixtures of hemoglobin and lipid or ink and lipid, which are preheated to the same temperature in 30 s. All measurements are performed within 30 min after the phantoms are made. The bottom layer is made by pouring the mixture of Hb, lipid, and gel into a plastic mold with a cross-sectional area of 3 × 3 cm^2^ and a depth of 4 cm (Figure 1a). The top layer is made by embedding the mixture of ink, lipid, and gel between two microscope glass slides, which are separated by a glass coverslip of thickness 0.20 mm. Lastly, the top layer is cut and transferred the top of the bottom layer to create a two-layer phantom (Figure 1b). In all the phantoms, the final concentrations of agar and lipid are 1% *w*/*v*, whereas final concentration of ink is 1% *v*/*v*. Here, PPG pulses are measured when the top layer thickness (*D*) is 0 and 0.20 mm.

Figure 1d,e summarize the optical properties of these phantoms. The reduced scattering (*μ*_s_′) of lipid 1% was extracted from our previous work [22]. To calculate the mean values and standard deviations (which are later shown as error bars), three separate sets of phantoms are made. This is equivalent to a total of 42 phantoms for all *D* values. The cost of chemicals, including agar, ink, blood, and Intralipid, used to fabricate these 42 phantoms is within USD 40. In addition, the volume of these phantoms satisfies the semi-infinite condition for photon migration. This is estimated by comparing the phantom dimensions to the diffusion theory’s effective penetration depth [29,30], which is approximately 0.1 cm at the lowest level of attenuation (wavelength of 900 nm) in this case.

### 2.2. Instrumentations: Broadband Reflectance Spectroscopy System

**Figure 2:**
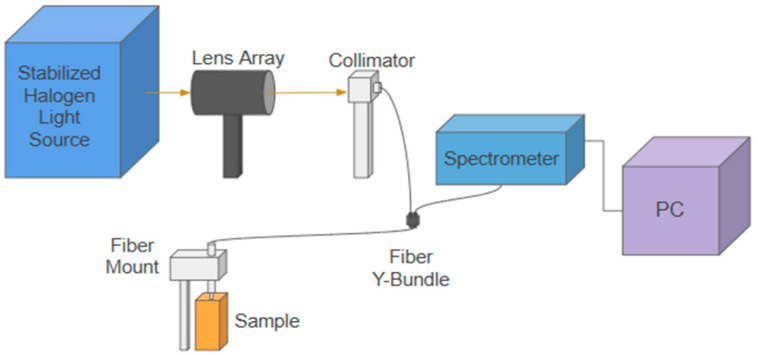
A schematic drawing of the customized reflectance spectroscopy system. The fiber probe is mounted normally on the sample surface. In all the phantom measurements, the fiber probe slightly touches the phantom’s surface. In the spectralon measurement, the fiber probe is positioned above the sample surface at a distance so that maximum intensity is collected at the spectrometer. An MATLAB v2024a graphical user interface is used to control the spectrometer and to collect and analyze the PPG pulses.

As shown in Figure 2, the customized reflectance spectroscopy system incorporates a stabilized quartz Tungsten-Halogen source (SLS302, Thorlabs, Newton, NJ, USA), a lens array (EF 75–300 mm, Thorlabs, Newton, NJ, USA), a collimator (PAF2S—A7A, Thorlabs, Newton, NJ, USA), a customized fiber optics bundle with a source–detector distance (SDD) of approximately 0.5 mm, a spectrophotometer (HDX-VIS-NIR, Ocean Optics Inc., Orlando, FL, USA), a fiber mount to hold the sample leg of the probe, and a PC with MATLAB v2024a software for instrument control and data analysis. Like the other studies [24,26,31,32,33,34], the diffuse reflectance signal (DRS) is calculated by dividing the reflected intensity collected with the detection fibers from the phantom to that from a Spectralon^®^ target of 99% reflectance factors in the wavelength region of 450–900 nm (Labsphere Inc., North Sutton, NH, USA). Unlike other studies [24,26,31,32,33,34], this paper does not focus on solving for optical properties, but rather on measuring the PPG pulses and studying their spectral dependence.

### 2.3. Monte Carlo Simulations

To support the spectral PPG trends observed in phantom measurement, as well as in the previous in vivo studies, Monte Carlo eXtreme (MCX) method [15] is used to compute both the PPG and mean photon pathlengths in the skin at wavelengths of 523 nm (green) and 945 nm (NIR). MCX is utilized for its compatibility with common graphic processing units (GPUs), computational speed, and abilities to simulate photon migration in complex biological media [35,36,37,38,39,40,41,42,43,44]. A previous model is utilized to simulate human skin consisting of the epidermis, dermis, and blood vessels in the dermis coded as cylinders [9]. The diameter of these cylinder is varied to simulate a PPG pulse. The optical properties for skin type 6 [3] are used in the simulations. To observe the relative trends (PPG as a function of wavelengths), the values of the simulated optical properties do not need to be the same as those of the phantoms because of the chromophores’ similar spectral shapes (i.e., blood, or ink vs. melanin, or Intralipid vs. tissue scattering). Epidermal thicknesses of 50, 100, and 150 µm are considered for the various body types [45,46,47,48,49,50,51,52,53,54], while detectors located at source–detector distances (SDD) of 1, 2, 3, and 4 mm are studied.

**Figure 3:**
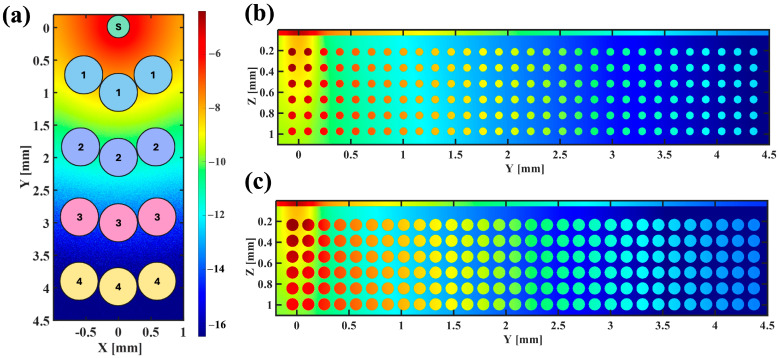
(**a**) A schematic of the source (S) and detectors on the surface of the tissue at SDD = 1, 2, 3, and 4 mm (center to center); (**b**,**c**) normalized energy fluence for the diastole (**b**) and the systole (**c**).

The number of photons launched is selected to achieve approximately 6 million photons detected for each wavelength at each SDD. A Gaussian source with a beam diameter of 0.4 mm and a series of three detectors with radii of 0.3 mm at each SDD are placed on the skin surface. Figure 3a shows a schematic for the placement of detectors relative to the source. The simulations are performed on an Dual GeForce RTX 2080 GPU (NVIDIA Co., Santa Clara, CA, USA) and take approximately 7.4 h to launch 28 billion photons when SDD = 4 mm. The simulated tissue volume is 2 × 4.7 × 1.1 mm^3^ (X-Y-Z), with a voxel size of 2 µm. As demonstrated in Figure 3, to simulate the AC and DC of the cardiac cycle, the vessels of two different diameters are simulated: 75 µm (systole) and 117 µm (diastole). The cross-sectional vessel density is 33.75 vessels/mm^2^, which is within the range found in the literature [55,56].

The mean optical pathlength (MOP) is calculated using Equation (1), where *i* represents the tissue layer, *j* represents the detected photon, *l* is the optical pathlength recorded with MCX, and *w* is the photon weight.

(1) $$\mathrm{MOP}=\left(\sum_{i}\sum_{j}l_{i,j}\;w_{i,j}\right)/\left(\sum_{i}\sum_{j}w_{i,j}\right)$$

The quality of the PPG signal is evaluated through the *AC/DC* ratio, which is calculated from the logarithms of the diffuse reflectance signal (*DRS*). This concept is illustrated in Equation (2), where the maximum (max) value corresponds to the diastolic phase and the minimum (min) value corresponds to the systolic phase.

(2) $$AC/DC=100\%\times \left[\max\left(-logDRS\right)-\min\left(-logDRS\right)\right]/\min\left(-logDRS\right)$$

## 3. Results

### 3.1. Phantom Measurement: PPG as a Function of Wavelength

**Figure 4:**
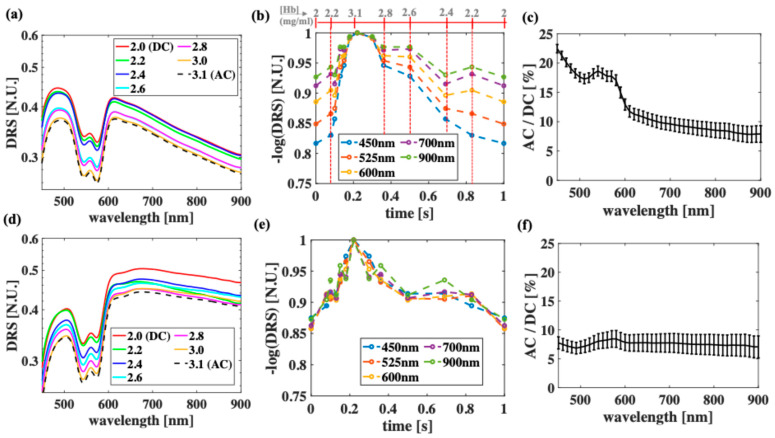
Experimental measurement of optical phantoms when (**a**–**c**) *D* = 0 and when (**d**–**f**) *D* = 0.20 mm. (**a**,**d**) Diffuse reflectance signal (DRS) as function of wavelengths when Hb concentration is varied from 2.0 to 3.1 mg/mL, (**b**,**e**) corresponding PPG waveform at selected wavelengths, (**c**,**f**) and AC/DC ratios. Due to current instrument limitation, all measurement was performed at SDD = 0.5 mm.

Figure 4 shows the averaged measurements from each of the three sets of optical phantoms when *D* = 0 (Figure 4a–c) and *D* = 0.20 mm (Figure 4d–f). Overall, the DRS decreases when the Hb concentration increases (Figure 4a,d), with some exceptions where the Hb concentrations are low and close to one another, introducing larger errors especially at long wavelengths. To obtain the PPG waveforms, the negative logarithms of DRS for each phantom are calculated and plotted as a function time (scaled accordingly to the Hb concentration) at the selected wavelengths (Figure 4b,e). As expected, the PPG signal improves when D increases (Figure 4b vs. Figure 4e), with a significant decrease at the short wavelengths. For example, *AC/DC* at 450 nm decreases about 67% when *D* increases from 0 to 0.20 mm. This number is less than 10% wavelength of 900 nm. In addition, the shorter wavelengths (400–500 nm) yield a higher *AC/DC* than the longer wavelengths (700–900 nm) at *D* = 0. This is because strong Hb absorption produces a high *AC/DC* for short wavelengths at *D* = 0, though this phenomenon becomes less effective when *D* increases. Moreover, at both the thicknesses, the wavelengths between 540 and 580 nm produce better PPG signals than the NIR wavelengths.

### 3.2. PPG vs. MOP at Green and NIR Wavelengths

**Figure 5:**
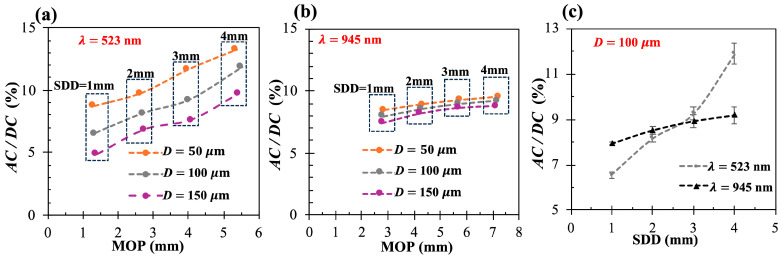
(**a**,**b**) *AC/DC* vs. MOP when epidermal thickness *D* = [50, 100, 150] µm and SDD = [1–4] mm at wavelengths of 523 nm (**a**) and 945 nm (**b**); (**c**) *AC/DC* as function of SDD when *D* = 100 µm at both wavelengths.

Figure 5 plots *AC/DC* as a function of the MOP when λ = 523 nm (Figure 5a) and λ = 945 nm (Figure 5b), considering the different *D* values and the different SDD values. Within each group of SDD, when the epidermal thickness increases from 50 to 150 µm, green PPG decreases significantly, while NIR PPG decreases slowly. As the SDD increases (MOP increases), green PPG increases significantly, while NIR PPG increases slowly. This phenomenon resembles NIRS studies of the brain, where brain sensitivity increases with the SDD [57]. However, when moving from green to NIR wavelengths, the trends may oppose. In particular, when SDD > 2 mm and *D* < 150 µm, green light produces a better PPG signal despite having a shorter *MOP* than that of NIR light. For example, when *D* = 100 µm and SDD = 4 mm, green *AC/DC* is 29% higher than NIR *AC/DC*, whereas the green *MOP* is 30% lower than the NIR *MOP*. At the thickest epidermis and SDD = 4 mm, NIR light performs no more than 8% better than green light.

## 4. Discussion and Conclusions

Using simple and cost-effective phantoms made of well-established chromophores, the PPG pulses are successfully simulated and measured at the two different top layer thicknesses. Although seven phantoms are used to demonstrate how a PPG pulse can be simulated, only two phantoms are needed (one at *AC* and one at *DC*) to calibrate the PPG signal (*AC/DC*). In addition, previous studies showed that the spectral shape of similar gel-based phantoms is stable over 2 weeks if stored at 4C degrees and can be reused [21], further demonstrating the cost-effectiveness of the current calibration method.

Both the phantom measurement (Figure 4) and the simulations (Figure 5) in this work further confirm that green PPG is better than NIR PPG when the epidermal thickness is small [8] and that increasing epidermal thickness significantly deteriorates the green PPG signal, while slightly deteriorating the NIR PPG signal. While the wavelengths within 520–540 nm are still within the green perception of normal optics nerves, the phantom measurement shows that wavelength of 540 nm may produce a better PPG signal than that of 523 nm due to the Hb peak at 540 nm. In addition, PPG at wavelength of 580 nm (a secondary Hb peak) may be useful, though it is well within the error bars of green PPG. These observations are in agreement with previous simulations on non-compressed skin model [58]. While the experimental measurements are limited to SDD = 0.5 mm, Monte Carlo simulations extend the understanding of similar observations to include longer SDD. Moreover, the simulations show that increasing the SDD improves the green PPG signal dramatically, while improving NIR PPG slightly. Though the phantom measurement is limited by several factors, including the wavelength region from 450 nm to 900 nm and a short SDD (0.5 mm), the experimental trends agree well with the simulated trends, and both demonstrate the clear advantage of shorter wavelengths over NIR wavelengths when studying PPG signals.

On the other hand, by comparing the simulated *AC/DC* and *MOP*, we can make qualitative suggestions to improve PPG in commercial wearable devices. While our previous studies offered the ultimate solution to improve heart rate monitoring with time-of-flight PPG [14], this study offers the following additional and simpler methods to improve steady-state continuous wave PPG: (1) increase the *MOP* by increasing the source–detector distance because blood determines the shape of the PPG pulse and is confined within vessels that are only found (or as simulated) in the dermis and (2) use a shorter wavelength, where blood absorption is more dominant. In future work, we will focus on re-designing the fiber probe to include larger SDDs (up to 4 mm), adding more spectrometers to allow for the simultaneous calculation of PPG at different SDDs and extend studies to in vivo measurement.

## Data Availability

The data are available upon reasonable request.
